# Poly(ethylene glycol)-*graft*-Hyaluronic Acid Hydrogels for Angiogenesis

**DOI:** 10.3390/polym17212845

**Published:** 2025-10-24

**Authors:** Miyu Hashimoto, Kazune Oda, Ari Yamamoto, Ik Sung Cho, Yasuhiko Tabata, Masaya Yamamoto, Tooru Ooya

**Affiliations:** 1 Department of Medical Device Engineering, Graduate School of Medicine, Kobe University, 7-5-1 Kusunoki-cho, Chuo-ku, Kobe 657-0017, Japan; 2 Department of Chemical Science and Engineering, Graduate School of Engineering, Kobe University, 1-1 Rokkoudai-cho, Nada-ku, Kobe 657-8501, Japan; 3 Institute for Materials Chemistry and Engineering, Kyushu University, CE41 744 Motooka, Nishi-ku, Fukuoka 819-0395, Japan; iksung_cho@ms.ifoc.kyushu-u.ac.jp; 4 Laboratory of Biomaterials, Department of Regeneration Science and Engineering, Institute for Life and Medical Sciences, Kyoto University, South Research Bldg. No. 1, 53 Kawara-cho Shogoin, Sakyo-ku, Kyoto 606-8507, Japan; tabata.yasuhiko.7n@kyoto-u.ac.jp; 5 Department of Materials Processing, Graduate School of Engineering, Tohoku University, 6-6-02 Aramaki-aza Aoba, Aoba-ku, Sendai 980-8579, Japan; masaya.yamamoto.b6@tohoku.ac.jp; 6 Center for Advanced Medical Engineering Research & Development (CAMED), Kobe University, 1-5-1 Minatojimaminami-machi, Chuuou-ku, Kobe 650-0047, Japan

**Keywords:** hyaluronic acid, hydrogels, Poly(ethylene glycol), growth factor, angiogenesis

## Abstract

Hyaluronic acid (HA) hydrogels are promising biomaterials for tissue engineering and drug delivery due to their biocompatibility and biodegradability. The objective of this study was to develop a novel HA-based hydrogel for the controlled release of basic fibroblast growth factor (bFGF) to promote angiogenesis. A series of PEG-grafted HA hydrogels with varying PEG grafting ratios were synthesized and characterized. We evaluated their physicochemical properties, including swelling ratio, cross-linking density, and enzymatic degradation behavior, and assessed their ability to control bFGF release and induce angiogenesis in a mouse model. The results showed that the PEG-grafting ratio significantly affected the gel properties. Notably, the PEG60-*graft*-HA hydrogel exhibited a higher swelling ratio and more rapid degradation, suggesting a non-uniform and highly porous structure. In vitro release studies confirmed that while PEG5-*graft*-HA and PEG15-*graft*-HA gels showed burst release, the PEG60-*graft*-HA hydrogel demonstrated sustained release of bFGF over time. Furthermore, in vivo experiments revealed a significant increase in angiogenesis with the PEG60-*graft*-HA hydrogel, likely due to the prolonged release of active bFGF. These findings suggest that PEG-grafted HA hydrogels, particularly those with a higher PEG grafting ratio, are promising biomaterials for the controlled release of growth factors and applications in tissue regeneration.

## 1. Introduction

In recent years, hydrogels have attracted significant attention in a wide range of applications, including tissue engineering, drug delivery, coatings, flexible electronics, and 3D printing, owing to their excellent flexibility, elasticity, high water content, and biocompatibility [1]. Hydrogels are defined as three-dimensional polymer networks containing a large amount of water, typically formed through physical, chemical, or covalent crosslinking. Research on functional hydrogels has expanded in multiple directions, and one example is the development of anisotropic conductive hydrogels. However, while such studies have mainly focused on imparting electrical or mechanical properties, the present study aims to develop a novel hydrogel based on hyaluronic acid (HA), a major component of the extracellular matrix, with a specific emphasis on the retention and controlled release of growth factors.

Hyaluronic acid (HA) is a major component of the extracellular matrix, a linear polysaccharide consisting of *N*-acetyl-D-glucosamine and D-glucuronic acid linked by β-glycosidic bonds to form repeating disaccharides with molecular weights ranging from 0.8 to 20,000 kDa [2]. HA is a mucopolysaccharide present in various tissues in the body and has been used clinically as a building block of biomaterials for over 30 years because of its immunoneutrality [3]. HA is a component involved in the migration and differentiation of cells. HA, which normally exists in vivo as a macromolecule >10^6^ Da, is hydrolyzed by the enzyme hyaluronidase, which cleaves the β-1,4 and β-1,3 bonds. This half-life can range from a few hours to several days [4]. High molecular weight HA exhibits anti-angiogenic and anti-inflammatory effects. On the other hand, low molecular weight HA (<3.5 × 10^4^ Da) exhibits proinflammatory and angiogenic properties [5,6]. High molecular weight hyaluronan is a major component of the extracellular matrix (ECM). In contrast, low molecular weight hyaluronan is generated in vivo through the enzymatic action of hyaluronidase [7], which is the main component of ECM. This low molecular weight HA is known to promote vascular endothelial cell proliferation and migration in vitro and angiogenesis in vivo [7] HA is found in all tissues and fluids of animals and is particularly abundant in early embryos, where it forms a pericellular coat around most cells and functions as a signaling molecule that interacts with binding proteins to regulate cell adhesion, migration, and proliferation [8]. HA is also found in the pericellular coats of most cells.

HA is also utilized as cross-linked gels. Chemical cross-linking is a stable cross-linking method that forms covalent bonds between functional groups. Chemical cross-linking can establish stable and irreversible hydrogel networks in the preparation of hydrogels for tissue engineering [9]. Generally, hydrogels are used in a variety of medical applications, including skin substitutes, adhesives, matrices for drug delivery, and scaffolds for tissue engineering [10]. For example, in 1992, Yui et al. evaluated the inflammation-responsive biodegradation of cross-linked HA gel. This suggested that cross-linked HA gels are promising as an inflammation-responsive degradable matrix for implantable drug delivery [11]. In 2012, Schramm et al. prepared hydrogels by UV photocrosslinking HA and *N*-vinylpyrrolidinone. This gel was suggested to be highly biocompatible and a potential alternative for long-term vitreous replacement [12]. Wang et al. created a cross-linked biomaterial using genipin, a natural compound that is mesoarticulated from the fruit of the beech tree. This biomaterial has sufficient mechanical strength and can promote chondrogenic differentiation of seeded cells, making it suitable as a cartilage material [9]. This cross-linked HA gel could result in a drug sustained release system [12]. Addition reactions, condensation reactions, and radical polymerization continue to be developed for hyaluronan hydrogels in biomedical applications. Addition and condensation reactions include thiol-modified hyaluronan, haloacetate-modified hyaluronan, aldehyde-modified hyaluronan, and Huisgen cycloaddition. Hydrogels prepared using these methods have been shown to be effective in cell and molecule delivery, as well as cell proliferation. Radical polymerization includes reactions with anhydrous methacrylic acid, glycidyl methacrylate, and hydrolyzable hyaluronan. These are thought to have applications in cartilage tissue regeneration, cardiac repair, molecular delivery, and microdevices [4].

Poly(ethylene glycol) (PEG) is a polymer compound composed of repeating ethylene oxide units. It is proposed from ethylene oxide, a three-membered ring ether, anionically ring-opened by alkali metals or hydroxides, and exists in a wide range of molecular weights from 200 to over 10,000 g/mol. It is a nonionic polymer with high biocompatibility. They also have a variety of structures, including PEGs with various functional groups at the ends and hyperbranched PEGs. It is approved and used by the U.S. Food and Drug Administration (FDA) in a variety of biochemical applications such as food, cosmetics, and formulations [13,14,15,16]. So far, hydrogels based on PEG, which has excellent biocompatibility, have been prepared and used as scaffold materials for 3D cell culture [17]. The use of PEG as a scaffold material for three-dimensional cell culture has been studied.

Growth factors include epidermal growth factor family (EGF), transforming growth factor-beta family (TGF-beta), fibroblast growth factor family (FGF), vascular endothelial growth factor (VEGF), granulocyte macrophage colony stimulating factor (GM-CSF), platelet derived growth factor (PDGF), connective tissue growth factor (CTGF), interleukin family (IL), and tumor necrosis factor-α-family [18]. In particular, growth factors that act on endothelial cells, smooth muscle cells, and pericytes, which are necessary for the formation of blood vessels, are called vascular growth-related growth factors. Fibroblast growth factor (FGF) was first extracted from bovine pituitary gland in 1973 and was able to be produced uniformly in 1983. The mammalian FGF consists of 22 species and is divided into seven subfamilies [19].

It is important to maintain the stability of the activity of the bioactive substance to be delivered. Since growth factors are proteins, they have a higher-order structure, and if this higher-order structure is disrupted even slightly, the bioactivity will be inactivated. Therefore, as an approach to maintain stability, heparin has been the focus of attention when using cell growth factors, which represent bioactive substances. Another example of maintaining protein stability is the molecular crowding effect of PEG. Molecular crowding refers to a change in the properties of molecules in a solvent when a high concentration of a protein or other macromolecule is present, and this molecular crowding is observed in cells. Therefore, by adding a high concentration of PEG to a protein solution, a crowding environment is simulated and protein stability is maintained [20]. The PEG solution is then added to the protein solution to simulate the crowding environment and maintain protein stability. Pike et al. reported in 2006 that a heparin-containing hyaluronic acid gel [21]. In this report, heparin content enabled long-term storage and release while retaining in vivo activity. In 2013, Nguyen et al. reported a heparin-mimetic polymer conjugated with bFGF [22]. The polymer was stable for 42 days and protected from degradation by heat, acids, and proteolytic enzymes, demonstrating retention of bioactive stability. These results indicate that controlled release and long-term storage are essential for drug delivery.

The easiest way to incorporate growth factors into hydrogels is to add them directly to the hydrogel. However, this method results in rapid burst release during the initial swelling phase. Therefore, controlling the release of growth factors over a long period of time is a challenge [23]. In addition, biodegradable systems for controlled release drug delivery are currently being widely investigated because they do not require invasive techniques such as surgery. However, drug diffusion can lead to serious side effects if a different drug than the one intended to be delivered is released from the matrix. In 1996, Moriyama et al. reported that a heterogeneously structured hydrogel of degradable dextran matrices containing PEG as a drug reservoir had been developed for a novel design of implantable device for peptide delivery [24]. In addition, the use of hyaluronic acid in place of the dextran matrix has been confirmed for controlled degradation and insulin release [25]. However, the size and distribution of these PEG domains were not controlled. Therefore, they prepared hydrogels with PEG-grafted dextran, which enabled them to control PEG. Therefore, in 1999, Moriyama and colleagues prepared hydrogels using PEG-grafted hyaluronic acid to control insulin release [25]. In particular, PEG and HA are known to form aqueous two-phase systems (ATPS), in which proteins preferentially partition into one of the phases [25]. This property suggests that grafting PEG onto HA can provide the ability to control protein distribution and retention. Moreover, compared with other chemical modifications of HA (e.g., esterification or amidation), PEG grafting offers advantages such as easier reaction control, improved solubility, and enhanced structural stability as well as molecular crowding. Therefore, PEG grafting is considered an effective strategy for achieving stable incorporation and controlled release of growth factors in HA-based hydrogels. However, there is still no example of controlled release of cell growth factors such as basic FGF (bFGF) encapsulated in a gel.

In this study, we propose a new hydrogel using PEG-grafted hyaluronic acid and bFGF, a cell growth factor, and how it is released into the gel. In detail, we tried to clarify a relationship between changing the grafting rate of PEG and the release behavior of bFGF. We also evaluated whether there is a correlation between the grafting rate and the degradability of the hydrogel. It is suggested that angiogenesis is influenced by the local concentration and biological activity of bFGF. Moreover, while high molecular weight HA alone does not appear to induce angiogenesis, HA oligosaccharides have been reported to promote angiogenesis in synergy with a growth factor. In addition, it has been indicated that the degradation behavior and mechanical properties of HA hydrogels can be modulated by changes in molecular weight, crosslinking degree, and chemical modifications, which may affect angiogenic activity in vivo [2,3]. Therefore, the use of PEG-grafted HA may provide a promising strategy to regulate three important factors for angiogenesis: bFGF concentration, bFGF bioactivity, and HA degradation. Furthermore, we evaluated the physiological activity of bFGF by subcutaneously implanting bFGF-added hydrogel in mice and confirming its angiogenic effect.

## 2. Materials and Methods

### 2.1. Reagents

Hyaluronic acid (HA) (*M_n_*: 230,000 g/mol) purchased from JNC Corporation, Tokyo, Japan Polyethylene Glycol (PEG) (*M_n_*: 6000 g/mol), Deuterium oxide (D_2_O), Sodium chloride, Fluorescein isothiocyanate, Isomer I (FITC), Sodium carbonate and Ammonium chloride were purchased from Nacalai Tesque Corporation, Kyoto, Japan. α-Methyl-ω-aminopropoxy-polyoxyethylene (PEG-NH_2_) (*M_n_*: 5205 g/mol), α-Aminopropyl-ω-aminopropoxy,-polyoxyethylene (PEG-BA) (*M_n_*: 2121 g/mol) were purchased from NOF Co., Tokyo, Japan. 4-(4,6-Dimethoxy-1,3,5-triazin-2-yl)-4-methylmorpholinium chloride (DMT-MM), Potassium dihydrogen phosphate, potassium chloride, disodium hydrogenphosphate, sodium chloride, phosphate pH standard equimolal solution, phthalate pH standard solution, 2,4,6-trinitrobenzenesulfonic acid sodium salt dihydrate (TNBS), hydrochloric acid, hyaluronidase, from Ovine Testes (EC 3.2.1.35) were purchased from Wako Pure Chemical Industries, Osaka, Japan. Fiblast spray was purchased from Kaken Seiyaku, Hyogo, Japan.

### 2.2. Experimental Apparatus and Equipment

The differential scanning calorimeter (DSC) was an EXSTRA6000 DSC6200 Differential Scanning Calorimeter and its accompanying analysis software from Seiko Instrument Inc. The lyophilizer was an EYELA FD-1000 model from Tokyo Rikakikai Co., Ltd. (Tokyo, JAPAN). The dialysis tubes were Fast Gene Regenerate Cellulose Dialysis Tubing FG DM P4-MWCO 12,000–14,000 from Japan Genetics Corporation (Tokyo, JAPAN). Scanning electron microscope (SEM) used was JEOL’s JSM-6510 in the Soft Matter Interfacial Chemistry Laboratory (Tokyo, Japan). A V-730 Spectrophotometer from JASCO Corporation (Tokyo, Japan) was used for absorbance measurements. Perkin Elmer LS55 from PerkinElmer Japan G.K. (Yokohama, Japan) was used for fluorescence intensity measurements and FL WINLAB SOFTWARE (Ver. 4.00.02) for analysis software. A confocal laser scanning microscope (CLSM) was a FluoVIewTMFV1000 Confocal Microscope from Olympus, Inc. (Tokyo, JAPAN). Nuclear Magnetic Resonance (NMR) was a 300 Hz FT-NMR apparatus (JNM-LA300 FT NMR system, JEOL Ltd., Tokyo, Japan). Size-exclusion chromatography (SEC) system was comprised with a pump (PU-980, JASCO Corporation, Tokyo, Japan) equipped with a 7.5 × 300 mm SEC column (GF-310 HQ, Showa Denko K.K., Tokyo, Japan), a light scattering detector (miniDAWN TriStar, Wyatt Technology Corporation, Santa Barbara, CA, USA), and a refractive index detector (Shodex RI-101 detector, Showa Denko, Tokyo, Japan). 

### 2.3. Synthesis of PEG-graft-HA

**Scheme 1:**
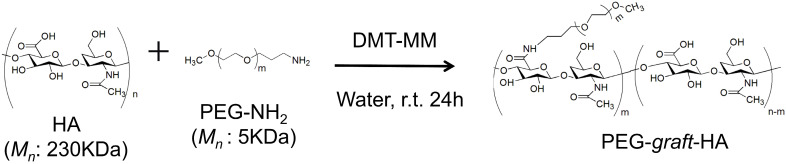
Synthesis of PEG-*graft*-HA.

HA (142 mg) was dissolved in 20 mL of distilled water, and PEG-NH_2_ was added at the specified amounts (the feed amounts and molar ratios are summarized in Table 1). DMT-MM (400 mg) was then added and allowed to react at room temperature for 24 h (Scheme 1). After the reaction was completed, the resulting mixture was dialyzed with distilled water for 2 days using a dialysis membrane (Spectra/Por^®^ 2, MWCO 12,000~14,000). After dialysis, the resulting solution was lyophilized for 24 h to obtain a white cotton-like solid. The obtained PEG-*graft*-HAs were characterized by ^1^H-NMR and SEC-multi-angle light scattering (MALS) measurements. The SEC-MALS measurements were operated at 37 °C under a flow rate of 0.5 mL min^−1^. D-PBS buffer was used as an eluent. The PEG grafting rate was quantified by DSC measurements (see; Section 2.4 and Section 2.5).

Yield: 87% ^1^H-NMR (300 MHz, D_2_O): δ = 1.85 (s, -NHCO-CH_3,_ 3H), δ = 2.76 (t, -CONH-CH_2_CH_2_CH_2_O-), δ = 3.22 (s, -[CH_2_CH_2_O]_m_-CH_3_, 3H), δ = 3.54 (s, -[CH_2_CH_2_O]_m_-,)

### 2.4. Evaluation of PEG Incorporation Rate Using DSC

The DSC measurement conditions are shown in Table S1. Samples were sealed in an aluminum pan and a reference αAlumina (5.48 mg) was used for the measurements under the following conditions: temperature range: −30 to 160 °C, temperature increase rate: 2 °C/min. All DSC measurements in this experiment were performed using the same method.

### 2.5. Calibration Curve for PEG Content

DSC measurements were performed on 5.0~7.5 mg of PEG-NH_2_ in aluminum pans under the conditions of Table S2. Calibration curves were generated from PEG-NH_2_ weight fractions and enthalpies of melting (n = 3 trials).

### 2.6. Calculation of PEG Incorporation Rate of PEG-graft-HA by DSC Measurement

A predetermined amount of PEG-grafted-HA shown in Table S3 was taken in an aluminum pan, and DSC measurements were performed under the conditions shown in Table S1 (n = 3). The PEG incorporation rate was calculated from the measured enthalpy of melting of PEG using the calibration curve shown in Figure S3.

### 2.7. Preparation and Characterization of HA, PEG-graft-HA Cross-Linked Gels

Conditions for gel preparation are shown in Table 2. The 5 wt% aqueous solution of HA was prepared, and 2 mL was added dropwise to a 35 mm dish. PEG-BA was added, and after stirring to ensure complete dissolution, DMT-MM was added and allowed to stand for 12 h at room temperature. After standing, the cross-linked gel was cut into pieces 1.0 cm in diameter and 2.0 mm thick. The gel was then immersed in distilled water for 2 days to remove unreacted material, during which time the distilled water was replaced. After the dialysis was completed, the gel was lyophilized to obtain a white solid.

The weight of the cross-linked gel after immersion in distilled water for 2 days (*W_wet_*) and the dry weight after lyophilization (*W_dry_*) were determined by weighing. These values were then used to calculate the swelling ratio (or water content) using the following Formula (1) (n = 3).

(1) $$Water\;content\;(\%)=\frac{W_{wet}-W_{dry}}{W_{wet}}\times 100$$

Observation of the gel after freeze-drying was performed by scanning electron microscopy (SEM). After freeze-drying, the cross-linked gel was cut into approximately 3.0 mm squares and adhered to a cover glass with pre-stretched carbon tape, and then platinum deposition was performed. SEM observation was performed at an acceleration voltage of 20 kV.

The weight of the gel after 6 days of immersion in 12 mM PBS solution (pH 7.4) was determined as *W_s_*_._ The swelling ratio was determined from Equation (2):

(2) $$Swelling\;ratio\;(\%)=\frac{W_{s}-W_{dry}}{W_{dry}}\times 100$$

**Scheme 2:**
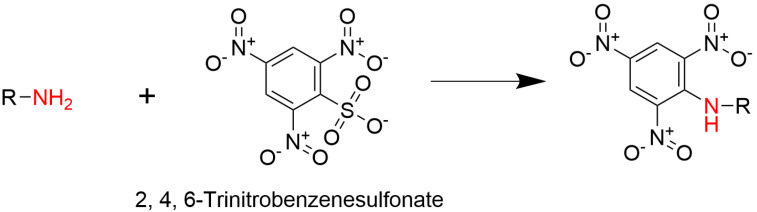
Mechanism of amino group determination with TNBS reagent.

The reaction rate of the crosslinker PEG-BA was determined from the quantitative method for amino groups using TNBS. In order to make a calibration curve, various concentration of PEG-BA was prepared (4.0 mL). To this solution, 2.0 mL of 0.1 wt% TNBS solution was added, and the solution was kept in a refrigerator for 1 day (see Table S4). Then, 300 μL of 2 M HCl solution was added to the solution to terminate the reaction, and the absorbance at 345 nm was measured by a spectrophotometer to obtain a calibration curve (Scheme 2). After measuring the volume of dialysis water of the crosslinked gels with distilled water, 4.0 mL of each dialysis water was weighed out and mixed with 2.0 mL of 0.1 wt% TNBS solution and left in a refrigerator for 1 day. 300 μL of 2M HCl solution was added to complete the reaction. The absorbance at 345 nm was measured using a spectrophotometer, and the amount of unreacted PEG-BA material in the dialysis water was calculated using the calibration curve obtained to calculate the crosslinker incorporation rate.

Autograph measurement and cross-linked gel measurement conditions were shown in Tables S5 and S6, respectively. The prepared crosslinked gels were cut into cylindrical shapes with a diameter of 1.0 cm and a thickness of 2.0 mm. The thickness (*t*_0_) and volume (*V*_0_) of the crosslinked gel before swelling were measured. After further swelling in 12 mM PBS solution (pH 7.4) until swelling equilibrium was reached, the thickness (*t_s_*) and volume (*V_s_*) of the cross-linked gel were measured. After wiping off the water on the surface and inserting the crosslinked gel between fixed plates, autographs were taken at a compression speed of 1.0 mm/min to measure the stress. The cross-linking density of the crosslinked gel was calculated by applying the results obtained to the theoretical formula for rubber elasticity shown in Equation (3) below (n = 5) [26]

(3) $$\tau =RT\frac{v_{s}}{V_{0}}v_{2}^{\frac{1}{3}}\left(\alpha -\frac{1}{\alpha^{2}}\right)$$

τ: Stress

R: Gas constant

T: Temperature

$\frac{v_{e}}{V_{0}}$: Cross-linking density

$v_{2}$: Inverse of the degree of swelling

α: Expansion/contraction ratio ($t/t_{0}$)

### 2.8. Evaluation of Enzymatic Degradation of PEG-graft-HA Cross-Linked Gels

The sample conditions are shown in Table S7. 12 mM PBS solution (pH 7.5) was used to swell the cross-linked gels for about 6 days and allowed to stand until swelling equilibrium was reached. The average thickness, surface area, and weight of the swollen cross-linked gels were then measured. 100 U/mL hyaluronidase solution was prepared in 12 mM PBS solution (pH 7.5). 5 mL of hyaluronidase solution (100 U/mL) was immersed in the cross-linked gel that had reached swelling equilibrium, and then the gel was shaken at 37 °C, 50 rpm. After each time elapsed, the crosslinked gel was shaken at 37 °C and 50 rpm. After each time, the cross-linked gels were removed from the hyaluronidase solution, the water in the cross-linked gels was lightly wiped off, and the weight was measured (n = 2, 3 trials). The hyaluronidase solution was changed every other day.

Based on the weight data obtained above, each was normalized according to the following Equation (4) to evaluate whether the degradation behavior of the cross-linked gel was surface degradation.

(4) $$M_{t}/M_{\infty }=t/t_{\infty }$$

$M_{t}$: Weight of gel after *t* hours of immersion in hyaluronidase solution

$M_{\infty }$: Total weight of gel

$t_{\infty }$: Time when the cross-linked gel is completely decomposed.

The remaining percentage of crosslinked gel was calculated according to Formula (5) to evaluate the degradability of the crosslinked gel.

(5) $$Hydrogel\;Remaining\;\left(\%\right)=\frac{W_{t}}{W_{0}}\times 100$$

$W_{t}$: Weight of gel after t hours of immersion in hyaluronidase solution

$W_{0}$: Weight of gel before soaking in hyaluronidase solution

### 2.9. Synthesis of FITC-labeld bFGF (FITC-bFGF)

To 500 μg of bFGF, 0.50 mL of distilled water was added and stirred. At this time, the mixture was still cloudy, but then 1 mL of 0.1 M Na_2_ CO_3_ (pH 11.54) was added to confirm complete dissolution. At this time, pH 12 was simultaneously confirmed. To this solution, 100 μL of 1 mg/mL FITC solution (in DMSO) was gently added in 10 μL drops. At this time, the DMSO and water were vortexed as needed to ensure adequate mixing. After 24 h of reaction in a refrigerator (at 4 °C), 1 mL of 5 M NH_4_Cl was added dropwise to ensure that pH 7 was reached. After stirring for another 2 h, the reaction was terminated. Then, gel filtration chromatography (Sephadex 25, expansion solvent: PBS(-)) was used to remove unreacted FITC to obtain the product. The solution of the product was lyophilized to remove water, and a yellow cottony solid was recovered. Since this synthesis was performed twice, the amount of FITC bound to each was calculated. A 20 mg/mL solution of the product was prepared and the amount of FITC bound was calculated by fluorescence intensity measurement (excitation wavelength: 488 nm, fluorescence wavelength: 530 nm).

### 2.10. Loading of FITC-bFGF into HA and PEG-graft-HA Hydrogels

FITC-bFGF was dissolved in PBS(-) to be 7500 ng/mL. Twenty or 6.7 μL of this solution was added to the HA hydrogel (150 or 50 ng/gel) and a leakage test was performed in 15 mL of PBS(-) after soaking in a refrigerator for 1 day.

### 2.11. Release Studies of FITC-bFGF Using HA and PEG-graft-HA Hydrogels

The procedure was performed in a 50 mL centrifuge tube with 15 mL of PBS(-) solution in a thermostatic chamber at 37 °C. Sampling times were 5, 10, 20, 30, 40, 50, 60, 120, and 180 min. Every hour, 0.70 mL of sample was squeezed out and the same volume of PBS(-) was added. The accumulated emission rate was calculated by measuring the fluorescence intensity (excitation wavelength: 488 nm, fluorescence wavelength: 530 nm) of 100 μL of the sample solution dropped onto a 96 well plate at n = 6. The cumulative emission rate was calculated using n = 4, omitting the maximum and minimum values from n = 6.

### 2.12. Evaluation of Angiogenesis in Mouse Dorsal Subcutaneous Tissue Using Hydrogels with Sustained bFGF Release

HA hydrogel, PEG5-*graft*-HA hydrogel, and PEG60-*graft*-HA hydrogel were used. One hundred μg of bFGF was added to these hydrogels. Animal experiments were conducted in full compliance with the “Guidelines for Animal Experiments at Kyoto University” and the relevant regulations of the Institute for Frontier Life and Medical Sciences, with utmost consideration for animal welfare. The research protocol was reviewed and approved by the Animal Welfare and Ethical Review Body (AWERB) at Kyoto University (Approval ID: F-148-22, 22 August 2015). Mice were housed under specific pathogen-free conditions with free access to food and water and monitored regularly for health and well-being. Humane endpoints were predefined, and mice showing severe suffering or >20% weight loss were euthanized using CO_2_ inhalation followed by cervical dislocation, in accordance with institutional protocols. Specifically, ddY mice (female, 6 weeks old, total 9 animals, n = 3 for each hydrogel) were anesthetized with Nembutal, and a small incision (approx. 5 mm) was made in the skin on their backs. Hydrogels impregnated with 100 µg of bFGF were implanted subcutaneously. One week after implantation, the mice were euthanized with a lethal dose of anesthetic, and the subcutaneous tissue (2 × 2 cm^2^) from the implantation site was harvested. Hemoglobin was then extracted from the harvested tissue by immersing it in a 17 mM Tris-HCl buffer (pH 7.6) containing 0.75% ammonium chloride at 4 °C for 24 h. The extracted hemoglobin was quantified using the Hemoglobin B-Test Wako kit (Wako).

The harvested subcutaneous tissue was fixed by immersing it in 4% paraformaldehyde/phosphate-buffered saline (pH 7.4) at 4 °C for 12 h. The solution was then replaced with 30% sucrose/phosphate-buffered saline, and the tissue was embedded using a Frozen Tissue Section Embedding Agent (OCT Compound, Sakura Finetek Japan, Tokyo, Japan). Frozen sections (approx. 5 µm thick) were prepared from the embedded tissue using a cryomicrotome (CM1950, Leica Microsystems, Tokyo, Japan) and stained with hematoxylin and eosin (H&E).

### 2.13. Statistical Analysis

Comparisons between two different groups were performed using Student’s *t*-test. Statistical differences among means were considered significant when *p* < 0.05. As for the data of the hemoglobin concentration, sample means were compared using the one-way ANOVA test. In the case of a significant difference between the means, multiple comparisons of the means were carried out using Fisher PLSD. Differences for which *p* < 0.05 was considered to be statistically significant. All the analysis was conducted using SPSS Statistics 21 (IBM Corp., Armonk, NY, USA).

## 3. Results and Discussion

### 3.1. Characterization of PEG-graft-HA

The PEG grafting of all the samples (Table 1) was characterized by ^1^H-NMR and SEC-MALS measurements. Typical ^1^H-NMR spectrum of 50PEG-*graft*-HA (see; Table 1) was shown in Figure S1. The peaks attributed to the acetyl groups of N-acetyl-D-glucosamine (d = 1.85 ppm), the terminal methoxy group of PEG (d = 3.22 ppm), and the repeated ethylene glycol units of PEG (d = 33.54 ppm). In addition, from the SEC-MALS chart of 50PEG-*graft*-HA, retention time of the peak top was 21.3 min (Figure S2a), while that of the mixture of HA and PEG-BA showed two peaks (peak tops: 22. 3 and 35.2 min) (Figure S2b). These results indicated that the PEG chain was covalently bound to the HA backbone.

### 3.2. PEG Incorporation Rate of PEG-graft-HA 

DSC measurements of HA showed no peak near 60 °C, where the melting enthalpy of PEG appears, suggesting that the peak near 60 °C in the DSC chart of the HA/PEG mixture or PEG-grafted-HA is derived from PEG. In fact, the peak around 60 °C in the DSC chart of PEG ($M_{n}$: 5000 g/mol) is semi-crystalline, and this crystalline part has a melting point of about 50 °C. Table S8 shows that there is a positive correlation between the PEG content weight ratio of the HA/PEG mixture and the magnitude of the enthalpy of fusion of PEG, suggesting that the PEG content ratio is related to the enthalpy of fusion of PEG. A calibration curve based on a linear curve between the PEG loading ratio and enthalpy of fusion was obtained (Figure S3). From the result, it can be concluded that the calibration curve obtained is applicable to the calculation of PEG incorporation rate in PEG-*graft*-HA.

The results of DSC measurement of PEG-*graft*-HA are shown in Table 3. Table 3 shows the results of calculating the PEG incorporation ratio of PEG-*graft*-HA using the calibration curve in Figure S3 based on the enthalpy of melting values calculated from the obtained DSC chart (Figures S4 and S5). Figure S6 and Table 4 shows the results of PEG-*graft*-HA PEG incorporation rate calculated using the calibration curve in Figure S3 based on PEG enthalpy values. Based on the slope of the calibration curve, the reaction rate of PEG-*graft*-HA was calculated to be about 73%, which is a high reaction rate.

### 3.3. Preparation of HA and PEG-grafted-HA Cross-Linked Gels

About 6 h after the preparation of the cross-linked gels, we checked whether the cross-linked gels had solidified by using a spatula. However, the PEG61-*graft*-HA crosslinked gel did not solidify, indicating that the gelation time may be slower than that of the other gels. Furthermore, the PEG61-*graft*-HA crosslinked gel was still soft after 12 h, suggesting that the crosslink density seems to be low.

### 3.4. Mechanical Evaluation of HA and PEG-graft-HA Cross-Linked Gels

Table 5 shows the measurement results of water content. The water content of the crosslinked gels was 98~99% for all the crosslinked gels, indicating that there was no difference regardless of the PEG incorporation ratio.

**Figure 1:**
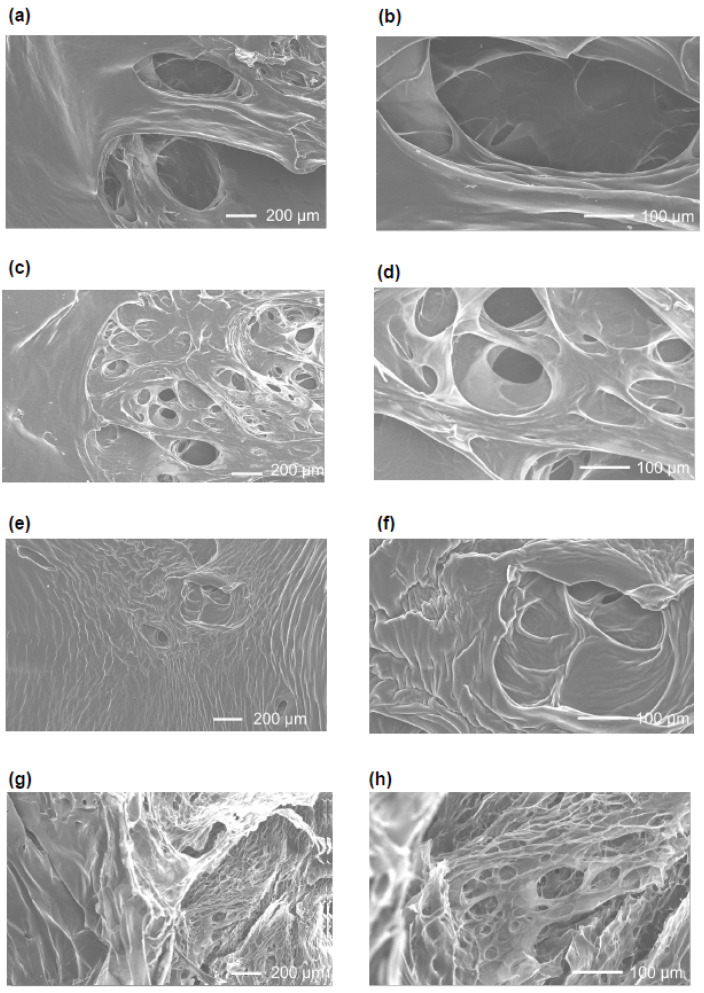
SEM images, they should be listed as (**a**) HA hydrogel (60× magnification); (**b**) HA hydrogel (200× magnification); (**c**) PEG5-*graft*-HA hydrogel (60× magnification); (**d**) PEG5-*graft*-HA hydrogel (200× magnification); (**e**) PEG15-*graft*-HA hydrogel (60× magnification); (**f**) PEG15-*graft*-HA hydrogel (200× magnification); (**g**) PEG60-*graft*-HA hydrogel (60× magnification); and (**h**) PEG60-*graft*-HA hydrogel (200× magnification).

SEM images of each gel are shown in Figure 1. The pore size of each gel was found to vary depending on the PEG incorporation ratio, with HA having a pore size of about 400 μm, 5 wt% PEG incorporation ratio 200 μm, 15 wt% 100 μm, and 60 wt% 100 μm, forming a porous network structure. PEG60-*graft*-HA crosslinked gel is more porous than other crosslinked gels.

**Figure 2:**
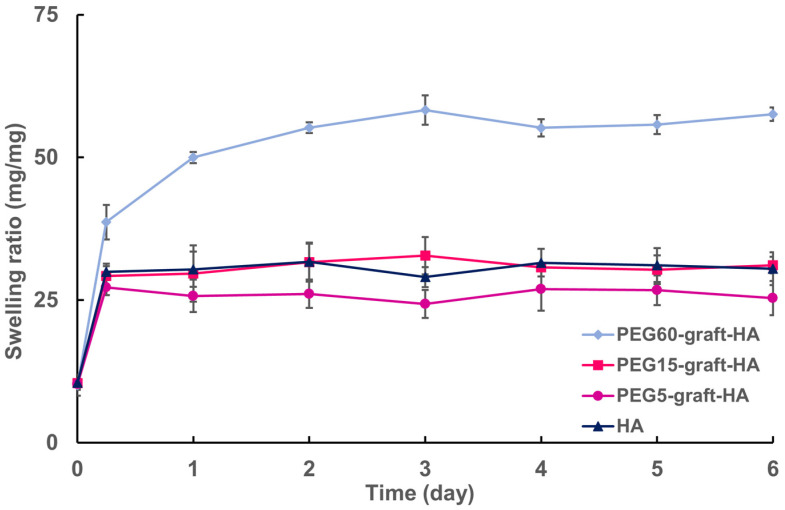
Swelling ratio change in the hydrogels as a function of time (n = 3, mean ± S.D).

Figure 2 shows the swelling ratio of each cross-linked gel measured over 6 days, plotted against time. For the PEG-*graft*-HA cross-linked gels with 5–15 wt%, the swelling ratio became constant one day after the onset of swelling. In contrast, the rate of increase in the swelling ratio for the PEG60-*graft*-HA cross-linked gel slowed after one day, becoming constant at approximately 60% after about 3 days.

**Figure 3:**
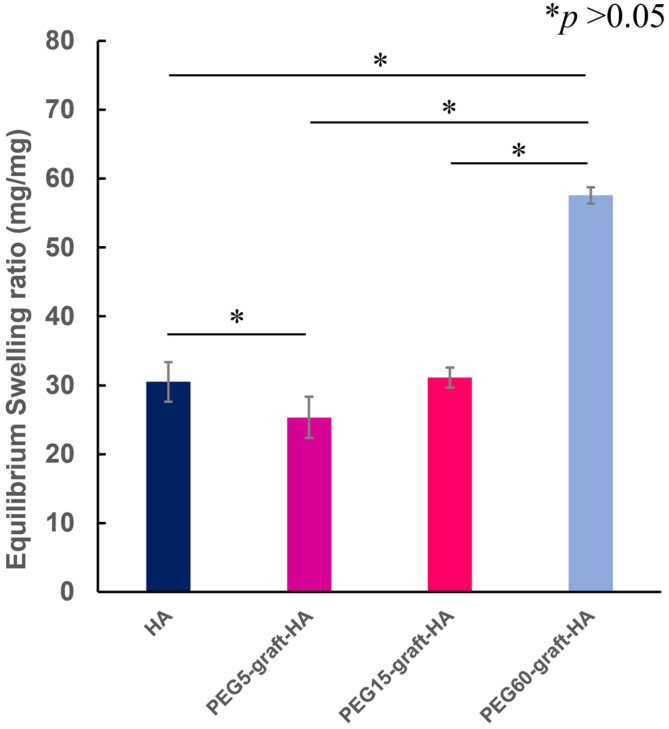
Equilibrium swelling ratio of the hydrogels (n = 3, mean ± S.D).

Figure 3 shows the swelling ratio 6 days after the onset of swelling, which is considered the point at which swelling equilibrium was reached. Six days after the onset of swelling, the PEG60-*graft*-HA cross-linked gel’s swelling ratio was approximately twice as high as the other cross-linked gels, showing a significant difference. This is attributed to the larger mesh size of the PEG60-*graft*-HA cross-linked gel, and the results of the swelling equilibrium measurements were also supported by SEM observations.

Figure S7 shows the calibration curve obtained from the absorbance measurement of the PEG-BA aqueous solution at 345 nm. Since the absorbance and concentration are in a proportional relationship, it was suggested that measurement is possible within this range.

The cross-linking introduction rate was calculated from the calibration curve shown in Figure S7, and the results are presented in Table S9. The results indicate that all samples showed a cross-linking introduction rate of over 95%.

**Figure 4:**
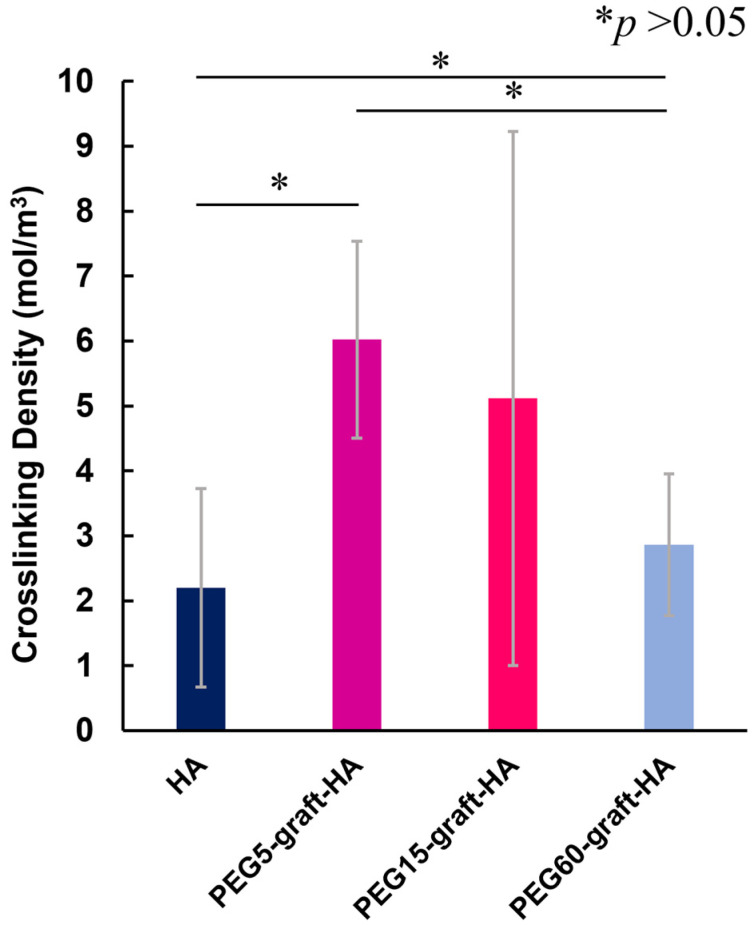
Crosslinking density of hydrogels (n = 3, mean ± S.D).

The crosslink density was calculated from the chart obtained from the autograph measurements (Figure S8) and the results are shown in Figure 4. The values were approximately 2.2 mol/m^3^ for the HA gel, 6.0 mol/m^3^ for the 5 wt% PEG-introduced gel, 5.1 mol/m^3^ for the 15 wt% gel, and 2.9 mol/m^3^ for the 60 wt% gel. Since the PEG5-*graft*-HA and PEG15-*graft*-HA gels showed no significant difference, their cross-linking densities are considered to be comparable. Furthermore, the cross-linking density of the PEG-*graft*-HA cross-linked gels with a 5–15 wt% PEG introduction rate increased by approximately 2 times compared to the HA cross-linked gel, and this result correlated well with the swelling ratio. Based on the results from Figure S4, where the cross-linking introduction rate was found to be similar across all cross-linked gels, it was suggested that the increased cross-linking density was due to the effect of physical cross-linking caused by the introduction of PEG. Moreover, even though the cross-linked gels with equal PEG introduction rates were prepared in the same 35 mm dish, a significant difference in cross-linking density was observed, suggesting that the cross-linking may have occurred non-uniformly.

While the PEG60-*graft*-HA cross-linked gel had a slightly lower cross-linking density than the HA cross-linked gel, its swelling ratio was approximately 2 times larger than the HA cross-linked gel. Therefore, no correlation was confirmed between the cross-linking density and the swelling ratio. It is believed that when the PEG60-*graft*-HA cross-linked gel reached swelling equilibrium and was compressed, the PBS solution inside the gel overflowed, making it unable to withstand the autograph measurement, and an accurate measurement of cross-linking density could not be achieved.

For this reason, a sufficient amount of water was removed from the PEG60-*graft*-HA cross-linked gel before the autograph measurement, which is why the calculated cross-linking density was not in a state of swelling equilibrium. Additionally, since the cross-linker introduction rate was similar to the other cross-linked gels, it is presumed that the cross-linker, PEG-BA, exists within the cross-linked gel by reacting at only one end due to the increased local concentration of the PEG graft chains.

### 3.5. Evaluation of Cross-Linked Gel Degradation Behavior

**Figure 5:**
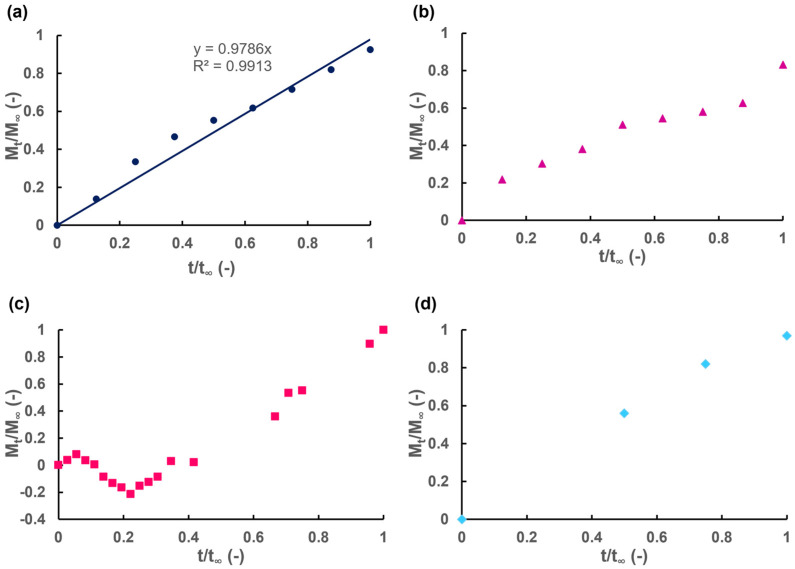
Degrading profile of hydrogel, they should be listed as (**a**) HA hydrogel; (**b**) PEG5-*graft*-HA hydrogel; (**c**) PEG15-*graft*-HA hydrogel; and (**d**) PEG60-*graft*-HA hydrogel.

Figure 5 shows the results for each cross-linked gel normalized by Equation (4). Based on the normalization using Equation (4), the HA cross-linked gel was determined to undergo surface degradation since a linear function with a slope close to 1 was obtained. In contrast, a linear correlation was not confirmed for the PEG-*graft*-HA cross-linked gels with 5 wt% and 15 wt% PEG introduction rates. This suggests that these gels may undergo bulk degradation rather than surface degradation. Furthermore, the degradation rate of the PEG60-*graft*-HA cross-linked gel was so fast that sufficient measurement data could not be obtained to determine if it was surface degradation.

**Figure 6:**
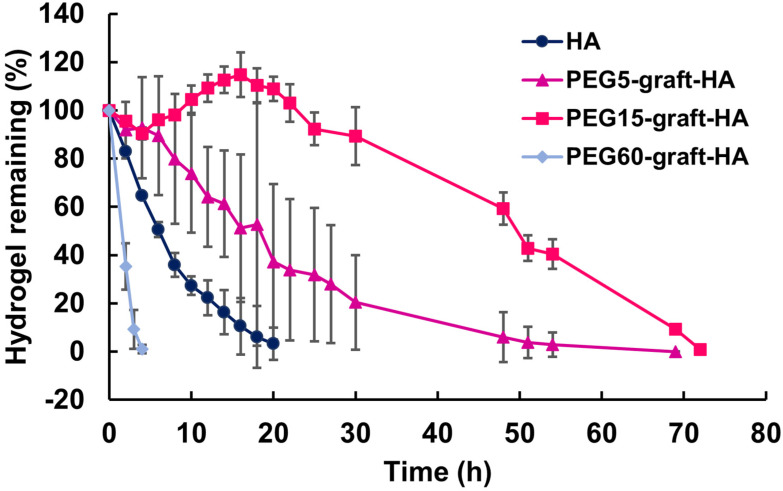
Degradation of hydrogels (Circle: HA hydrogel, Triangle: PEG5-*graft*-HA hydrogel, Square: PEG15-*graft*-HA hydrogel, Diamond: PEG60-*graft*-HAhHydrogel) in the presence of 100 U/mL of hyaluronidase in 12 mM PBS (pH 7.5) (n = 3, mean ± S.D).

Next, the remaining mass percentage of the cross-linked gels was calculated. Figure 6 shows the degradation behavior of each cross-linked gel obtained using Equation (5). The HA cross-linked gel’s degradation behavior, shown in Figure 5, and its decreasing remaining mass, shown in Figure 6, both confirmed that surface degradation occurred. The calculation of the remaining mass also showed that the PEG60-*graft*-HA cross-linked gel had the fastest degradation rate. From Figure 4, the PEG60-*graft*-HA cross-linked gel’s cross-linking density is significantly lower compared to the other gels. This suggests that the low cross-linking density may allow hyaluronidase to penetrate the gel. Comparing the degradation times of the HA cross-linked gel and the PEG60-*graft*-HA cross-linked gel, which have similar cross-linking densities, the PEG60-*graft*-HA cross-linked gel’s degradation rate was approximately 20% faster than the time it took for the HA cross-linked gel to completely degrade. This difference is likely influenced by the size of the gel after swelling. According to Table S7, the contact surface area between the hyaluronidase solution and the PEG60-*graft*-HA cross-linked gel is approximately 1.6 times larger than that of the HA cross-linked gel, which is thought to contribute to the increased degradation rate. Additionally, Figure 2 shows that the swelling ratio of the PEG60-*graft*-HA cross-linked gel is about twice that of the HA cross-linked gel. This may allow hyaluronidase to penetrate more easily into the gel. Therefore, it is believed that the degradation rate increased because both surface and internal degradation by hyaluronidase occurred simultaneously. Figure 4 also shows a correlation between the higher cross-linking densities of the PEG5-*graft*-HA and PEG15-*graft*-HA cross-linked gels and their lower degradation rates compared to the other gels.

Comparing the remaining mass of the HA cross-linked gel with the PEG5-*graft*-HA and PEG15-*graft*-HA cross-linked gels, Figure 6 shows that the HA gel’s remaining mass consistently decreased. In contrast, the PEG5-*graft*-HA and PEG15-*graft*-HA gels’ remaining mass increased from 7 to 18 h and then began to decrease. This behavior suggests that bulk degradation is occurring in the PEG-*graft*-HA gels. It is thought that hyaluronidase penetrates areas of low cross-linking density within the gel and hydrolyzes the HA, which further decreases the cross-linking density and causes the gel to swell.

Approximately 30 h after immersion in the hyaluronidase solution, these gels did not swell, and their remaining mass began to decrease. Thus, it was found that after about 30 h, the degradation behavior of the cross-linked gels changed from bulk degradation to surface degradation. This result suggests that the cross-linking density of the gels is non-uniform, with regions of low density where hyaluronidase can penetrate and regions of high density that are inaccessible to the enzyme.

Comparing the increase in the remaining mass of the PEG5-*graft*-HA and PEG15-*graft*-HA cross-linked gels, the increase was significantly more pronounced in the PEG15-*graft*-HA cross-linked gel. This suggests a higher degree of non-uniformity in its internal cross-linking density, leading to greater degradation as hyaluronidase penetrates the interior.

### 3.6. Characterization of FITC-bFGF

A calibration curve using FITC is shown in Figure S9. The results show that FITC can be measured in the range of 0 to 300 ng/mL; the fluorescence intensity of the FITC-bFGF solution was measured, and the FITC concentration was calculated from the fluorescence intensity. 90.2 ng of FITC was found to be present in 1.0 mg of FITC-bFGF. Assuming a 1:1 binding of FITC and bFGF, 150 ng or 50 ng of bFGF was added to the hydrogel.

### 3.7. Investigation of Loading Amount and Method of FITC-bFGF Using HA Hydrogel

To evaluate the effect of loading volume and loading time on the leakage behavior, a leakage test was performed 1 day after FITC-bFGF internalization, as shown in Figure S10. As a result, the release rate could be suppressed to 20~40%. Therefore, it is suggested that more than 1 day of immersion is necessary to encapsulate bFGF.

### 3.8. FITC-bFGF Release from HA and PEG-graft-HA Hydrogels

**Figure 7:**
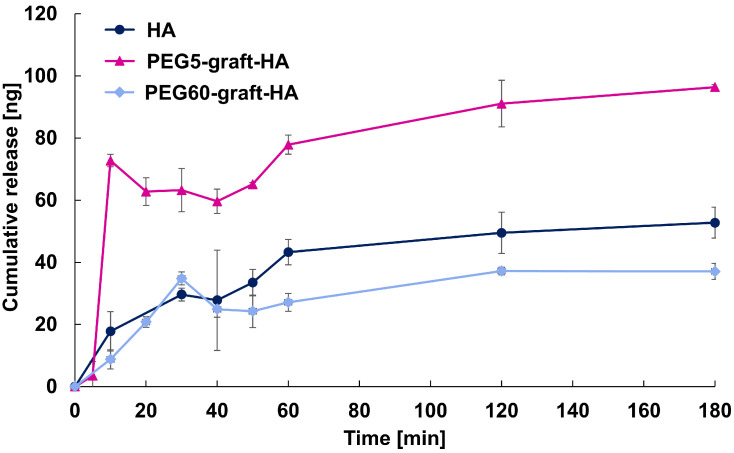
Profiles of FITC-bFGF release from FITC-bFGF-loaded hydrogels (Circle: HA hydrogel, Triangle: PEG5-*graft*-HA hydrogel, Diamond: PEG60-*graft*-HA hydrogel). The loading time was 24 h. The loading amount bFGF of FITC-bFGF was 150 ng/gel (n = 4, mean ± S.D.).

Figure 7 shows the release behavior of 150 ng of bFGF from HA hydrogels, PEG5-*graft*-HA hydrogels, and PEG60-*graft*-HA hydrogels. For the PEG5-*graft*-HA hydrogel, a burst release was observed just 10 min after the release began. In contrast, the release behavior of the PEG60-*graft*-HA hydrogel changed approximately 30 min after the start of the release, suggesting that the gel had swelled by that time. In addition, these results also suggests that increased amount of the PEG graft in the hydrogels led to suppress bFGF release, In order to discuss how the aqueous two phase system (APTS) of PEG and HA [24] affect the bFGF release, the partitioning behavior of fluorescein isothiocyanate-labeled basic fibroblast growth factor (FITC-bFGF) in a PEG-HA ATPS was examined. Stock solutions of 40 wt% PEG (*M_n_*: 6000 g/mol) and 10 wt% HA (*M_n_*: 230,000 g/mol) (prepared in PBS, pH 7.4) were prepared. To form the two-phase system, 0.5 mL of the PEG solution and 0.4 mL of the HA solution were mixed in an Eppendorf tube. A volume of 0.1 mL of an FITC-bFGF solution, containing a total of 750 ng of bFGF, was then added. The mixture was agitated with a spatula for complete homogenization and subsequently centrifuged for 5 min. Following centrifugation, the system was allowed to settle at room temperature.

The results, observed one day later and presented in Figure S11, showed a pronounced tendency for FITC-bFGF to partition into the PEG phase. Given that the isoelectric point (pI) of bFGF is 9.6, it was anticipated that the positively charged protein (pH 7.4) would partition into the negatively charged HA phase due to electrostatic attraction. The preferential partitioning into the PEG phase, contrary to this expectation, indicates the absence of substantial electrostatic interactions between HA and bFGF under these conditions. The slow release behaviors of the PEG-*graft*-HA hydrogels were considered to be due to bFGF partition into the PEG micro phase in the swollen hydrogels.

Furthermore, the release behavior observed in this study is consistent with previously reported findings on HA- and PEG-based hydrogels: HA-based hydrogels typically exhibit a pronounced initial burst release, and the rapid release observed within 10 min [3], which corresponds well to this trend with the case of the PEG5-g-HA hydrogel. In contrast, hydrogels with a high degree of PEG graft are known to retain proteins more stably and to release them in a sustained manner in response to swelling or degradation [27]. This is in agreement with our observation that the PEG60-g-HA hydrogel exhibited a change in release behavior after approximately 30 min, suggesting a transition toward sustained release. Taken together, these comparisons highlight that adjusting the PEG grafting ratio enables the design of release patterns ranging from rapid initial burst to swelling-mediated sustained release. Thus, our findings provide important insights into the strategic control of local concentration and duration of action of bFGF.

### 3.9. Evaluation of Angiogenesis in Hydrogel Under the Back Skin of Mice

**Figure 8:**
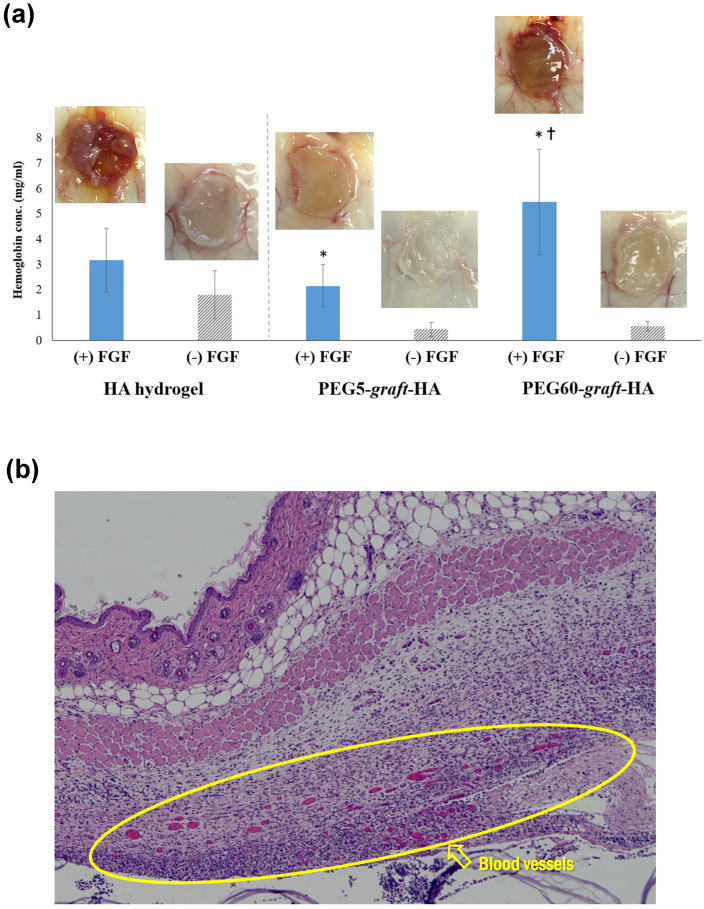
(**a**) Vasculation after 1 week (n = 3, Mean ± SEM) (†: *p* < 0.05 vs. the other samples bFGF (+), *: *p* < 0.05 vs. bFGF (−) in the same hydrogels, One-way ANOVA + Fisher PLSD), (**b**) an image of H&E-stained sections of specimens in the subcutaneous tissue implanted with the PEG60-*graft*-HA hydrogel containing bFGF after one week.

Figure 8a shows the results of the hemoglobin quantification. When bFGF was added into each hydrogel, the hemoglobin concentration increased by 1.8 times (HA hydrogel), 5.1 times (PEG5-*graft*-HA hydrogel) and 9.8 times (PEG60-*graft*-HA hydrogel). These results suggest that bFGF slow release on the implanted sites was contributed to the increased hemoglobin concentration. Since the pictures of Figure 8a showed good correlation between the increased hemoglobin concentration and the increased blood colors, the increased hemoglobin concentration should be due to angiogenesis. Also, the results indicate a significant increase in angiogenesis with the PEG60-*graft*-HA hydrogel. Comparing this with the results in Figure 7, it is possible that with the PEG5-*graft*-HA hydrogel, the bFGF was released so quickly after implantation that it either became inactive or the release stopped before it could promote angiogenesis. Conversely, with the PEG60-*graft*-HA hydrogel, the bFGF was released in a sustained manner over a longer period, which is believed to have successfully promoted angiogenesis.

Figure 8b shows an image of H&E-stained sections of specimens in the subcutaneous tissue implanted with the PEG60-*graft*-HA hydrogel containing bFGF after one week. A lot of blood vessels were observed, so that the PEG60-*graft*-HA hydrogel containing bFGF was a good candidate for inducing angiogenesis in vivo. Therefore, the findings of this study provide a valuable design strategy for developing hydrogels that can effectively promote chronic wound healing and tissue regeneration [28,29].

## 4. Conclusions

In this study, we successfully developed PEG-grafted HA hydrogels with varying PEG grafting ratios to address the challenge of burst release of growth factors. Our findings demonstrate a strong correlation between the PEG grafting ratio and the physicochemical properties of the gels. The PEG60-*graft*-HA hydrogel, with its higher swelling capacity and more rapid, bulk-like degradation, showed an ability to achieve sustained release of bFGF. This sustained release, in turn, resulted in a significant promotion of angiogenesis in a mouse subcutaneous model.

While we did not find a direct correlation between cross-linking density and swelling ratio, likely due to measurement limitations with the highly swollen gels, the overall data points to a key conclusion: the introduction of a high ratio of PEG grafts creates a unique gel structure that can effectively modulate the release kinetics of encapsulated proteins. This controlled, sustained release is crucial for maintaining the bioactivity of growth factors like bFGF, which is likely due to aqueous two-phase partition of bFGF into PEG region, and maximizing their therapeutic effect in vivo. Our results suggest strong potential for applications in regenerative medicine, particularly in vascular tissue engineering. However, several translational challenges remain, including:

- Stability of bFGF: While PEG grafting improves release control, long-term stability in vivo needs further improvement.
- Immunogenicity: Both bFGF and the hydrogel components may pose immunological risks that require thorough evaluation.
- Scalability: The synthesis and loading processes must be optimized for consistent large-scale manufacturing.

In the future, this material holds strong potential for clinical applications, particularly in improving therapeutic outcomes for diabetic foot ulcers, which affect approximately 18.6 million individuals worldwide each year. By accelerating wound healing and reducing the risk of amputation, this approach could offer significant social and medical benefits.

## Figures and Tables

**Table 1:** Conditions of PEG-*graft*-HA Preparation.

Sample	PEG Feed Ratio	HA		PEG-NH_2_		DMT-MM		Water	Recovered Amount	Efficiency
	(wt%)	(mg)	(nmol)	(mg)	(μmol)	(mg)	(μmol)	(mL)	(mg)	(%)
5PEG-*graft*-HA	5	141.2	613.9	7.5	1.4	110.8	400.4	20	125.8	84.6
10PEG-*graft*-HA	10	142.0	617.4	15.7	3.0	110.4	399.0	20	142.0	90.0
20PEG-*graft*-HA	20	142.2	618.3	36.4	7.0	110.0	397.5	20	150.4	84.2
35PEG-*graft*-HA	35	141.5	615.2	76.6	14.7	111.7	403.7	20	190.2	87.2
50PEG-*graft*-HA	50	142.1	617.8	142.1	27.3	110.5	399.3	20	249.1	87.6
75PEG-*graft*-HA	75	142.0	617.4	426.1	81.9	109.7	396.4	20	500.6	88.1
83PEG-*graft*-HA	83	141.7	616.1	668.3	128.4	110.5	399.5	20	667.8	82.4

**Table 2:** Value of PEG-*graft*-HA for DSC Samples.

Sample	PEG-*graft*-HA or HA	PEG-BA		DMT-MM		Water
	(mg)	(mg)	(μmol)	(mg)	(μmol)	(mL)
HA	100.4	199.9	94.2	99.1	358.1	2.0
PEG5-*graft*-HA	104.8	199.5	94.1	99.1	358.1	2.0
PEG15-*graft*-HA	117.2	200.8	94.7	99.6	359.9	2.0
PEG60-*graft*-HA	250.1	199.6	94.1	99.3	358.8	2.0

**Table 3:** DSC Results of PEG-*graft*-HA.

Sample	PEG Feed Ratio	ΔH (mJ/mg)				Standard Deviation
	(wt%)	n = 1	n = 2	n = 3	n = 4	
5PEG-*graft*-HA	5.0	1.38	1.15	1.95	1.49	0.41
10PEG-*graft*-HA	10.0	7.29	7.74	7.62	7.55	0.23
20PEG-*graft*-HA	20.4	15.65	15.65	19.78	18.27	2.27
35PEG-*graft*-HA	35.1	40.21	40.21	42.88	42.19	1.73
50PEG-*graft*-HA	50.0	63.53	65.53	67.62	66.14	2.26
75PEG-*graft*-HA	75.0	88.21	88.21	86.84	88.28	1.47
83PEG-*graft*-HA	83.0	96.03	96.03	98.73	97.09	1.44

**Table 4:** Calculated Results of PEG Ratio of PEG-*graft*-HA.

Revised Sample Name	PEG Feed Ratio	Calculated PEG Ratio
	(wt%)	(wt%)
PEG1-*graft*-HA (5PEG-*graft*-HA)	5	1
PEG5-*graft*-HA (10PEG-*graft*-HA)	10	5
PEG11-*graft*-HA (20PEG-*graft*-HA)	20	11
PEG26-*graft*-HA (35PEG-*graft*-HA)	35	26
PEG40-*graft*-HA (50PEG-*graft*-HA)	50	40
PEG54-*graft*-HA (75PEG-*graft*-HA)	75	54
PEG61-*graft*-HA (83PEG-*graft*-HA)	83	61

**Table 5:** Water Content of Hydrogels.

Sample	Water Content
	(%)
HA hydrogel	98 ± 0.4
PEG5-*graft*-HA hydrogel	98 ± 0.2
PEG15-*graft*-HA hydrogel	98 ± 0.2
PEG60-*graft*-HA hydrogel	98 ± 0.2

## Data Availability

The original contributions presented in this study are included in the article/supplementary material. Further inquiries can be directed to the corresponding author.

## Supplementary Materials

The following supporting information can be downloaded at https://www.mdpi.com/article/10.3390/polym17212845/s1:Table S1: DSC Measurement Conditions; Table S2: Mixture Ratio of PEG-NH_2_ and HA for DSC Samples; Table S3: Value of PEG-*graft*-HA for DSC Samples; Table S4: Conditions of PEG-BA aq; Table S5: Conditions for Autograph Measurement; Table S6: Conditions of Hydrogels for Autograph Measurement; Table S7: Conditions of Hydrogels for Degrading Assay; Table S8: DSC Results of Mixture of PEG-NH_2_ and HA; Table S9: Reaction Ratio of Crosslinking Agent (PEG-BA); Figure S1: Typical example of 1H-NMR spectrum of 50PEG-*graft*-HA (see; Table 1); Figure S2: SEC-MALS charts of (a) 50PEG-*graft*-HA (see; Table 1) and (b) a mixture of HA $(M_{n}$: 230,000) and PEG-BA ($M_{n}$: 2121). The SEC-MALS measurements were carried out, comprizing with a pump (PU-980, JASCO, Tokyo, Japan) equipped with a 7.5 × 300 mm SEC column (GF-310 HQ, Showa Denko K.K., Tokyo, Japan), a light scattering detector (miniDAWN TriStar, Wyatt Technology Corporation, Santa Barbara, CA), and a refractive index detector (Shodex RI-101 detector, Showa Denko, Tokyo, Japan) and operatiing at 37 °C under a flow rate of 0.5 mL min^−1^. D-PBS buffer was used as an eluent; Figure S3: PEG calibration curve of PEG-NH_2_ and HA mixture; Figure S4: DSC Curve Mixture((a) PEG-NH_2_:HA=1:0, (b) PEG-NH_2_:HA=7:1, (c) PEG-NH_2_:HA=2:1, (d) PEG-NH_2_:HA=1:1, (e) PEG-NH_2_:HA=1:2, (f) PEG-NH_2_:HA=1:5, (g) PEG-NH_2_:HA=0:1); Figure S5: DSC Curve of PEG-*graft*-HA ((a) 5PEG-*graft*-HA, (b) 10PEG-*graft*-HA, (c) 20PEG-*graft*-HA, (d) 35PEG-*graft*-HA, (e) 50PEG-*graft*-HA, (f) 75PEG-*graft*-HA, (g) 83PEG-*graft*-HA); Figure S6: Relationship between PEG feed ratio and PEG ratio in PEG-*graft*-HA; Figure S7: Calibration curve of the concentration of PEG-BA aq; Figure S8: Autograph Chart ((a) HA hydrogel, (b) 5PEG-*graft*-HA hydrogel, (c) 15PEG-*graft*-HA, (d) 35PEG-*graft*-HA, (e) 60PEG-*graft*-HA); Figure S9: Calibration curve of concentration of FITC; Figure S10: Profiles of FITC-bFGF release from FITC-bFGF loaded HA hydrogels; Figure S11: Results of distribution of FITC-bFGF in PEG/HA two phase system.
