# Supplementary material for: Poly(ethylene glycol)-graft-Hyaluronic Acid Hydrogels for Angiogenesis
Source: Polymers (Basel). 2025 Oct 24;17(21):2845. doi: 10.3390/polym17212845 (PMC12608165; doi:10.3390/polym17212845)
Supplement: Supplementary file 1 [file polymers-17-02845-s001.zip › Supporting information_SI_Polymers2.pdf]

Supporting information

Poly(ethylene glycol)-*graft*-hyaluronic acid hydrogels for angiogenesis

Miyu Hashimoto[1], Kazune Oda[2], Ari Yamamoto[2], Ik Sung Cho[3], Yasuhiko Tabata[4], Masaya Yamamoto[5], and Tooru Ooya [1,2,6*]

[1] Graduate School of Medicine, Department of Medical Device Engineering, Kobe University, 7-5-1 Kusunoki-cho, Chuo-ku, Kobe 657-0017, Japan; 249m115m@stu.kobe-u.ac.jp

[2] Graduate School of Engineering, Department of Chemical Science and Engineering, Kobe University,
1-1 Rokkoudai-cho, Nada-ku, Kobe 657-8501, Japan; 180t414t@gsuite.kobe-u.ac.jp (K.O.); 145t474t@gsuite.kobe-u.ac.jp (A.Y.)

[3] Institute for Materials Chemistry and Engineering, Kyushu University, CE41 744 Motooka, Nishi-ku, Fukuoka 819-0395, Japan; iksung_cho@ms.ifoc.kyushu-u.ac.jp

[4] Laboratory of Biomaterials, Department of Regeneration Science and Engineering, Institute for Life and Medical Sciences, Kyoto University, South Research Bldg. No. 1, 53 Kawara-cho Shogoin, Sakyo-ku,
Kyoto 606-8507, Japan; tabata.yasuhiko.7n@kyoto-u.ac.jp

[5] Graduate School of Engineering, Department of Materials Processing, Tohoku University, 6-6-02 Aramaki-aza Aoba, Aoba-ku, Sendai 980-8579, Japan; masaya.yamamoto.b6@tohoku.ac.jp

[6] Center for Advanced Medical Engineering Research & Development (CAMED), Kobe University, 1-5-1 Minatojimaminami-machi, Chuuou-ku, Kobe 650-0047, Japan

* Correspondence: ooya@tiger.kobe-u.ac.jp; Tel.: +81-78-308-2605

* Correspondence: ooya@tiger.kobe-u.ac.jp; Tel.: +81-78-308-2605

Table S1. DSC Measurement Conditions

Reference	Alpha Alumina
pan	Aluminum pan
Measured temperature	-30~160°C
Programming rate	2°C/min
Circumstance	Under a nitrogen gas

Table S2. Mixture Ratio of PEG-NH_2 and HA for DSC Samples

**Sample**	**Value (mg)**	**PEG-NH_2 (PEG) (mg)**	**HA (mg)**	**PEG ratio (wt%)**
PEG-NH_2 : HA=1:0	5.50	5.50	0.00	100.00
PEG-NH_2 : HA=7:1	6.57	5.72	0.85	87.06
PEG-NH_2 : HA=2:1	5.77	3.75	2.02	64.99
PEG-NH_2 : HA=1:1	8.25	4.09	4.16	49.58
PEG-NH_2 : HA=1:2	5.66	1.94	3.72	34.28
PEG-NH_2 : HA=1:5	6.35	1.11	5.24	17.48
PEG-NH_2 : HA=0:1	4.51	0.00	4.51	0.00

Table S3. Value of PEG-*graft*-HA for DSC Samples

**Sample**	**Value (mg)**
5PEG-*graft*-HA	5.00
10PEG-*graft*-HA	7.40
20PEG-*graft*-HA	5.55
35PEG-*graft*-HA	6.50
50PEG-*graft*-HA	4.90
75PEG-*graft*-HA	6.31
83PEG-*graft*-HA	7.50

Table S4. Conditions of PEG-BA aq

PEG-BA		Water	0.1 wt%TNBS
(μg)	(μg/mL)	(mL)	(mL)
0.0	0.0	4.0	2.0
250.0	62.5	4.0	2.0
390.0	97.5	4.0	2.0
490.0	122.5	4.0	2.0
520.0	130.0	4.0	2.0

Table S5. Conditions for Autograph Measurement

Assay	Compression
Shape of Hydrogels	cylinder
Compression Speed (mm/min)	1.0
Temperature (K)	296±1

Table S6. Conditions of Hydrogels for Autograph Measurement

Sample	Average Thickness (mm)		Average Volume (mm^3)	
	t_0	t_s	V_0	V_s
HA	2.0	2.0	157.1	388.8
PEG5-*graft*-HA	2.0	2.0	157.1	265.5
PEG15-*graft*-HA	2.0	2.8	157.1	447.9
PEG60-*graft*-HA	2.0	4.6	157.1	906.8

Table S7. Conditions of Hydrogels for Degrading Assay

Sample	Weight	Thickness	Dimension
	(mg)	(mm)	(mm^2)
HA	516.2	3.2	176.7
PEG5-*graft*-HA	333.8	3.0	124.5
PEG15-*graft*-HA	375.4	3.2	120.7
PEG60-*graft*-HA	808.0	3.6	287.6

Table S8. DSC Results of Mixture of $PEG-NH_2$ and HA

Sample	PEG ratio (wt%)	ΔH n=1	ΔH n=2	ΔH n=3	Average	Standard Deviation
$PEG-NH_2$: HA=1:0	100.00	168.60	162.09	161.85	164.18	3.83
$PEG-NH_2$: HA=7:1	87.06	145.68	134.97	112.15	130.93	17.13
$PEG-NH_2$: HA=2:1	64.99	104.14	111.67	110.00	108.60	3.95
$PEG-NH_2$: HA=1:1	49.58	84.8	82.28	81.75	82.84	1.45
$PEG-NH_2$: HA=1:2	34.28	54.32	53.42	49.18	52.31	2.75
$PEG-NH_2$: HA=1:5	17.48	25.63	24.27	24.69	24.69	0.82
$PEG-NH_2$: HA=0:1	0.00	0.00	0.00	0.00	0.00	0.00

Table S9. Reaction Ratio of Crosslinking Agent (PEG-BA)

Sample	Reaction Ratio (%)
HA	99.2
PEG5-*graft*-HA	97.5
PEG15-*graft*-HA	98.3
PEG60-*graft*-HA	97.7

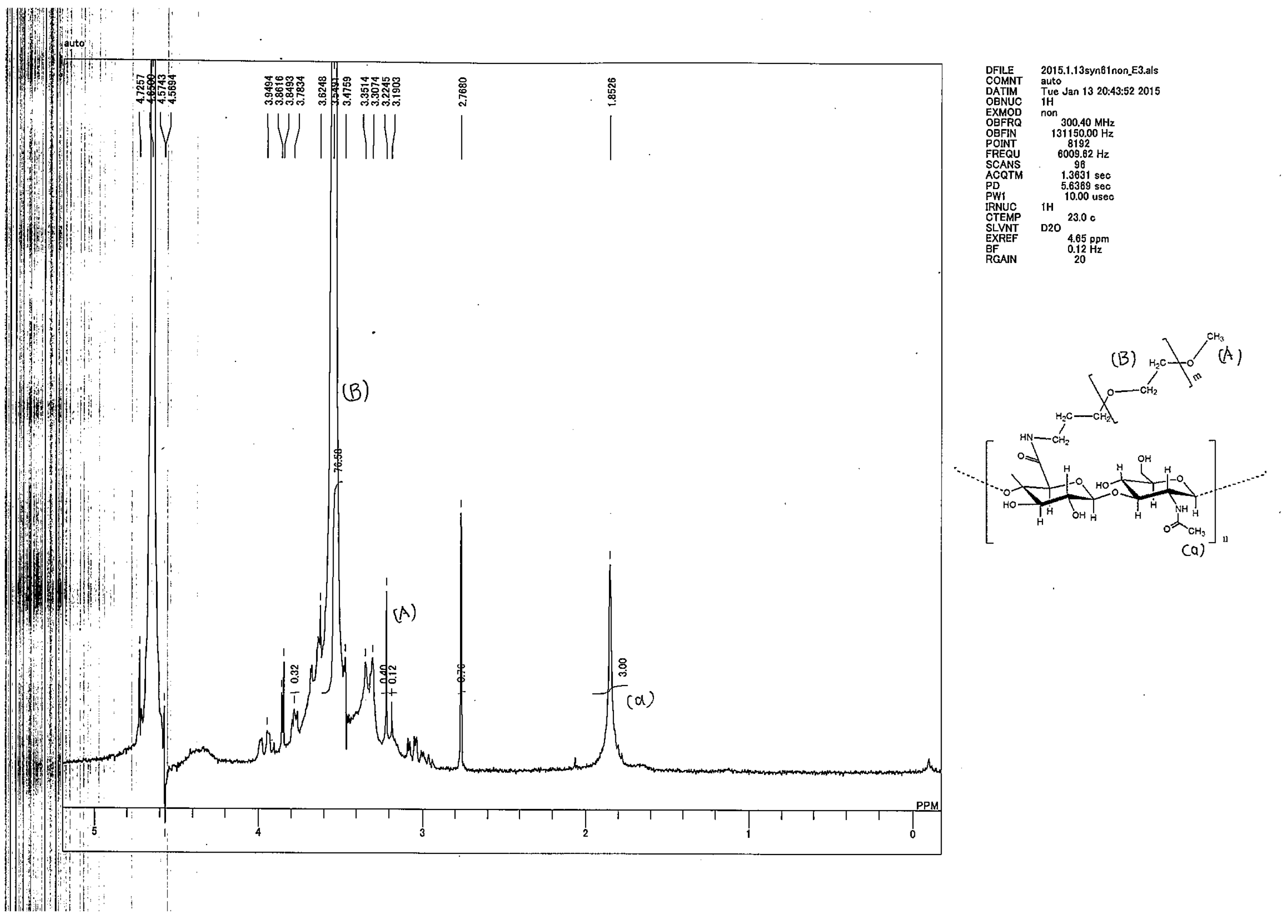

Figure S1. Typical example of ^{1}H-NMR spectrum of 50PEG-*graft*-HA (see; Table 1).

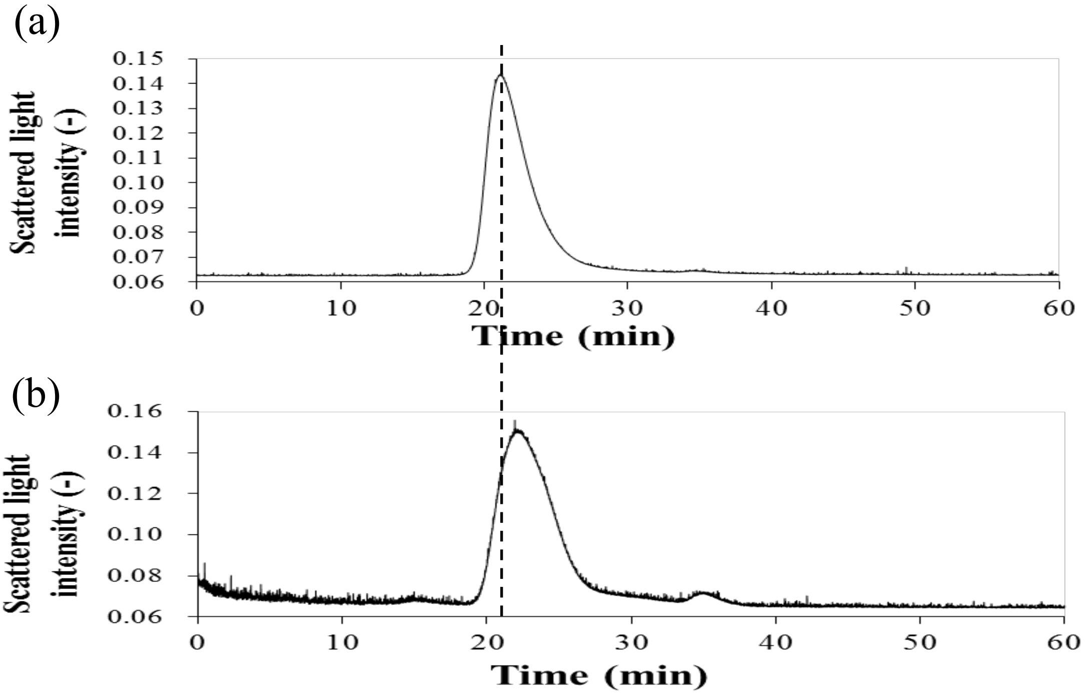

Figure S2. SEC-MALS charts of (a) 50PEG-*graft*-HA (see; Table 1) and (b) a mixture of HA (M_n : 230,000 g/mol) and PEG-BA (M_n : 2,121 g/mol). The SEC-MALS measurements were carried out, comprising with a pump (PU-980, JASCO, Tokyo, Japan) equipped with a 7.5 × 300 mm SEC column (GF-310 HQ, Showa Denko K.K., Tokyo, Japan), a light scattering detector (miniDAWN TriStar, Wyatt Technology Corporation, Santa Barbara, CA), and a refractive index detector (Shodex RI-101 detector, Showa Denko, Tokyo, Japan) and operating at 37 °C under a flow rate of 0.5 mL min^{-1}. D-PBS buffer was used as an eluent.

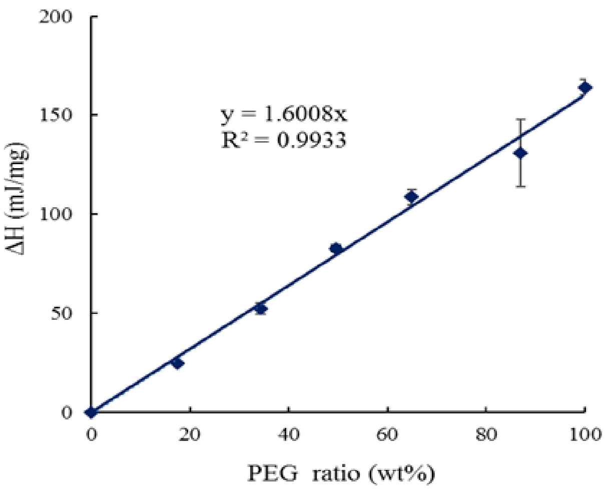

Figure S3. PEG calibration curve of PEG-NH_2 and HA mixture

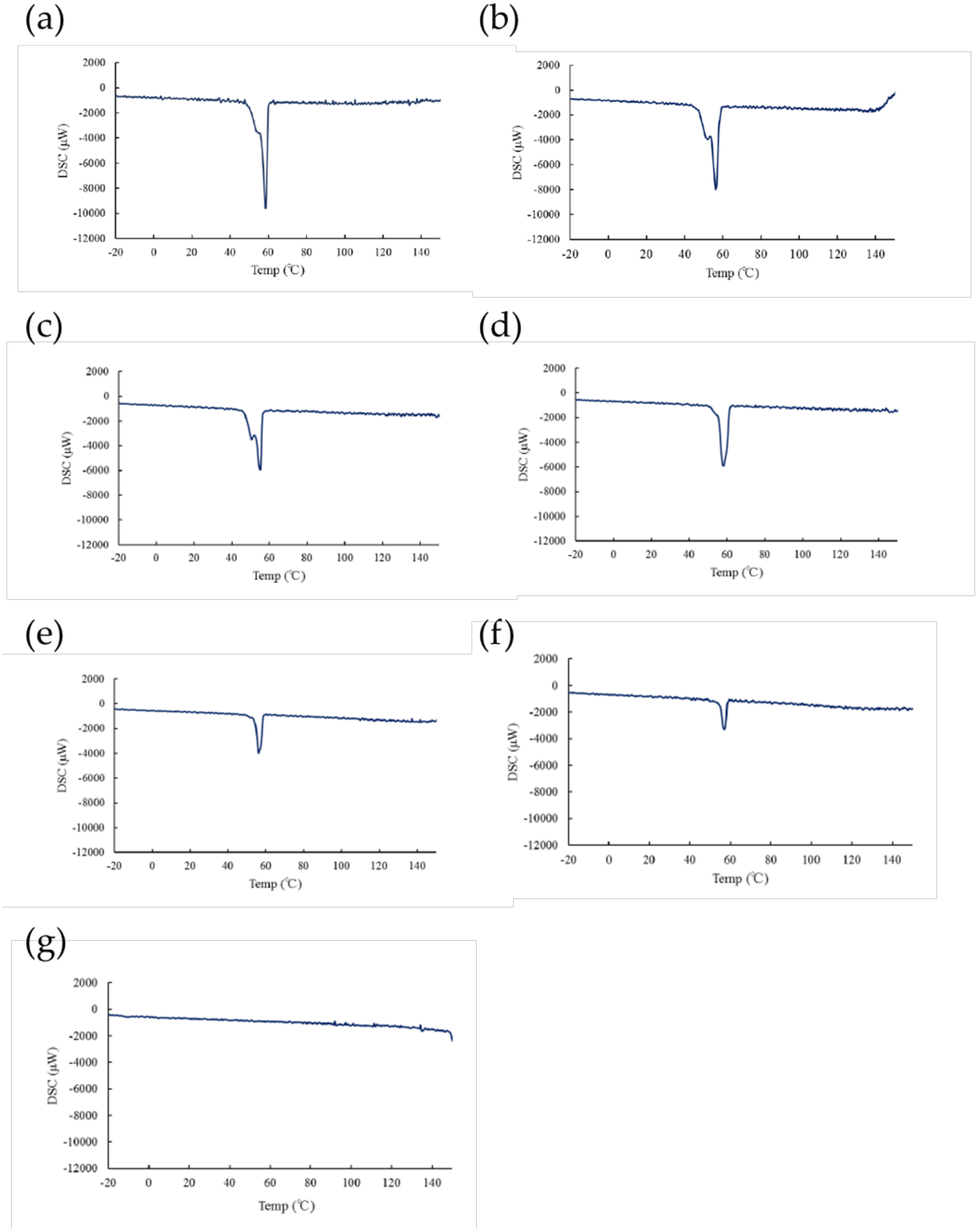

Figure S4. DSC Curve Mixture((a) PEG-NH_2:HA=1:0, (b) PEG-NH_2:HA=7:1, (c) PEG-NH_2:HA=2:1, (d) PEG-NH_2:HA=1:1, (e) PEG-NH_2:HA=1:2, (f) PEG-NH_2:HA=1:5, (g) PEG-NH_2:HA=0:1)

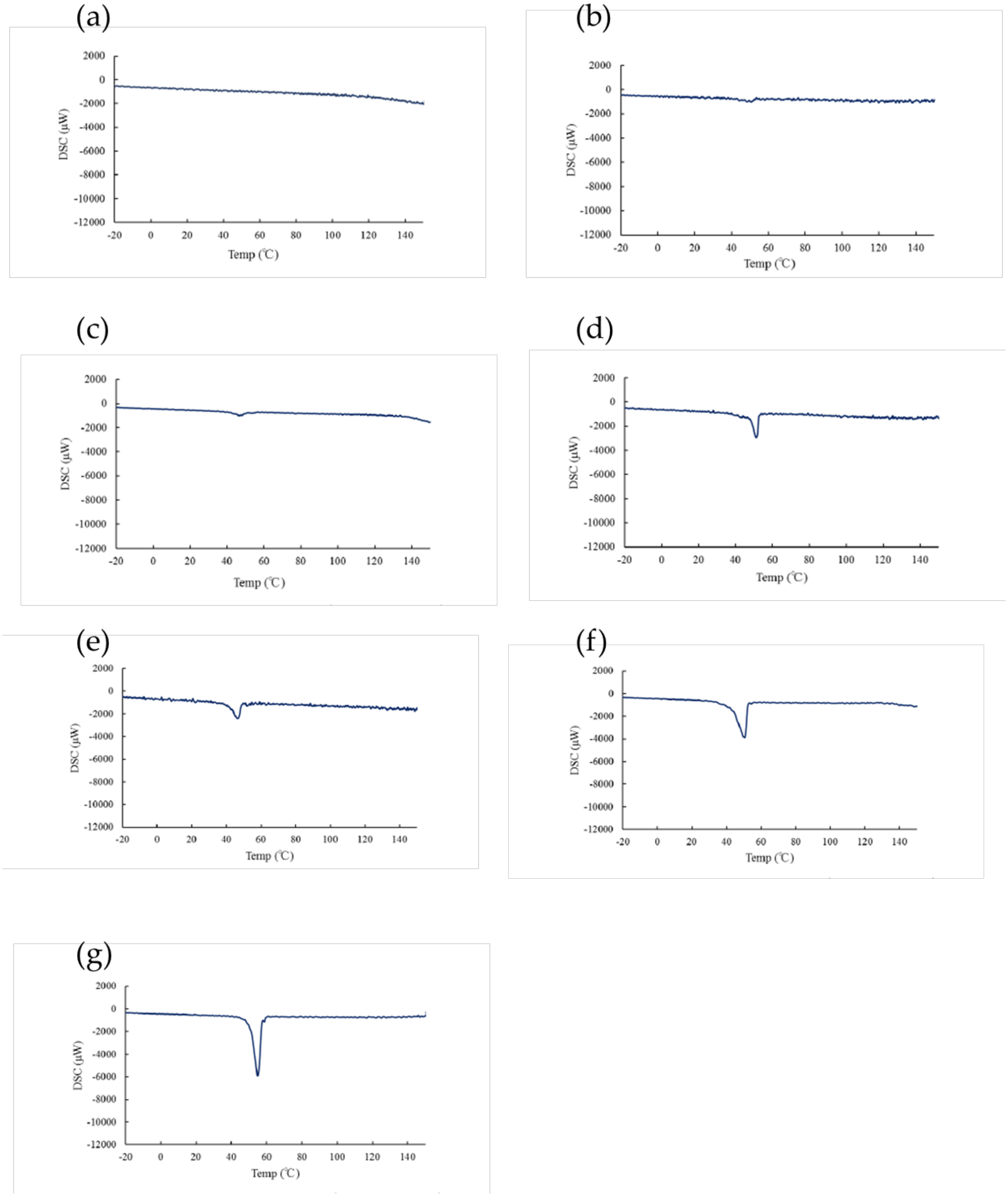

Figure S5. DSC Curve of PEG-*graft*-HA ((a) 5PEG-*graft*-HA, (b) 10PEG-*graft*-HA, (c) 20PEG-*graft*-HA, (d) 35PEG-*graft*-HA, (e) 50PEG-*graft*-HA, (f) 75PEG-*graft*-HA, (g) 83PEG-*graft*-HA)

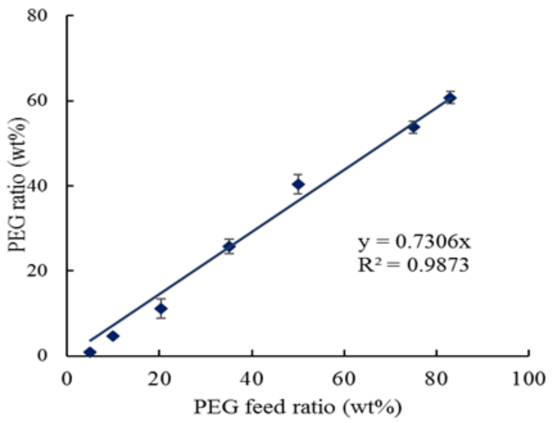

Figure S6. Relationship between PEG feed ratio and PEG ratio in PEG-*graft*-HA

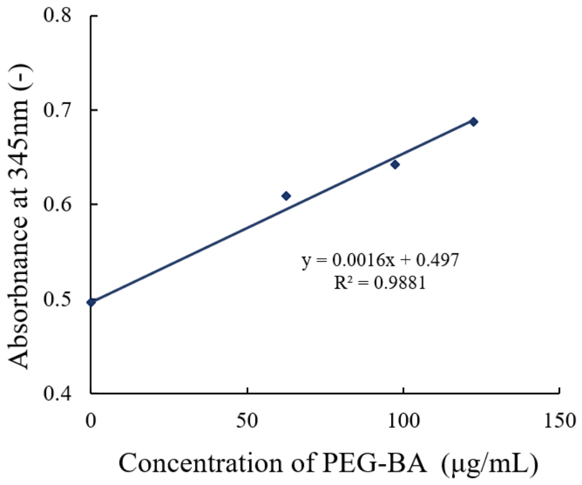

Figure S7. Calibration curve of the concentration of PEG-BA aq

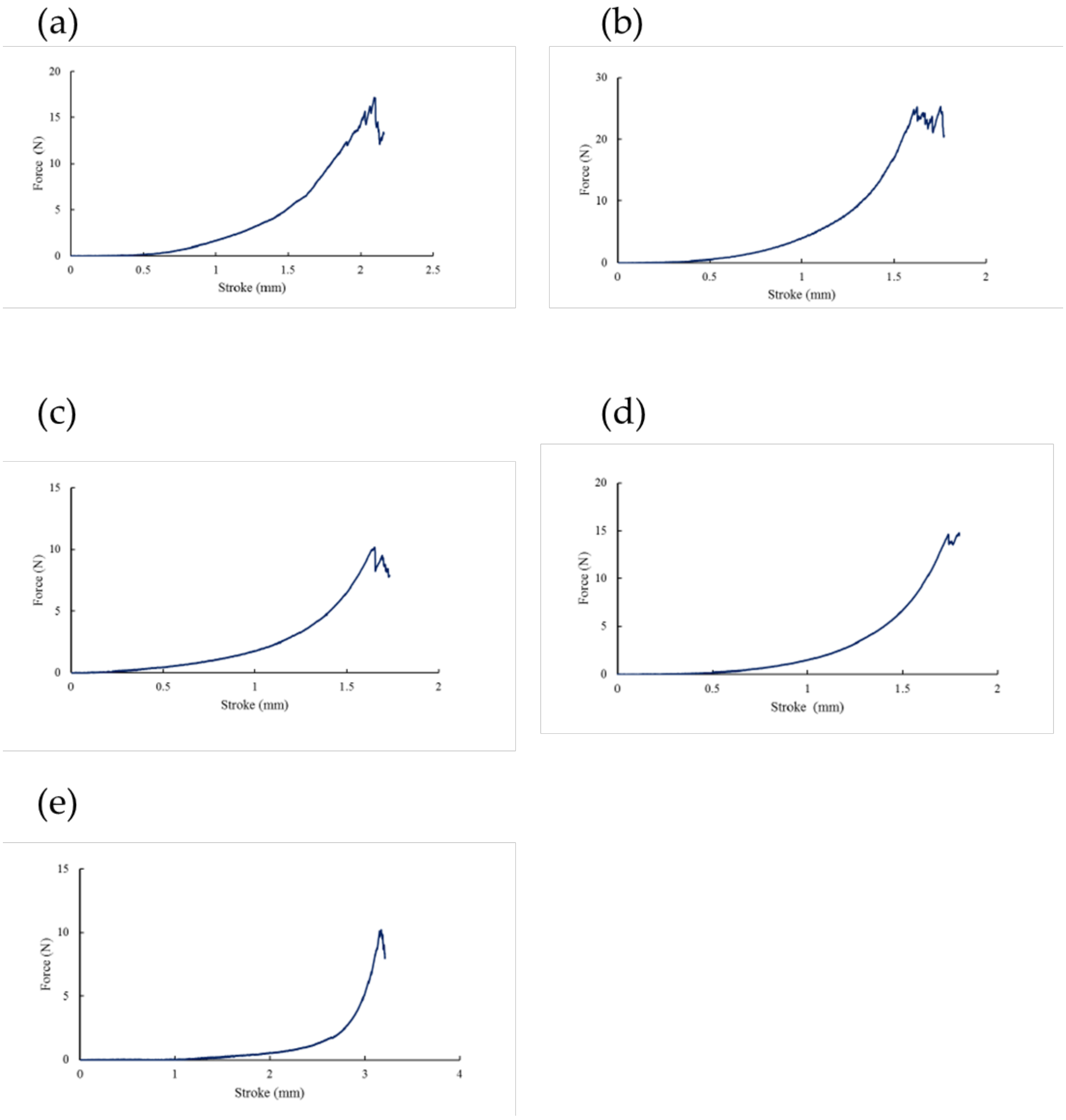

Figure S8. Autograph Chart ((a) HA hydrogel, (b) 5PEG-*graft*-HA hydrogel, (c) 15PEG-*graft*-HA, (d) 35PEG-*graft*-HA, (e) 60PEG-*graft*-HA)

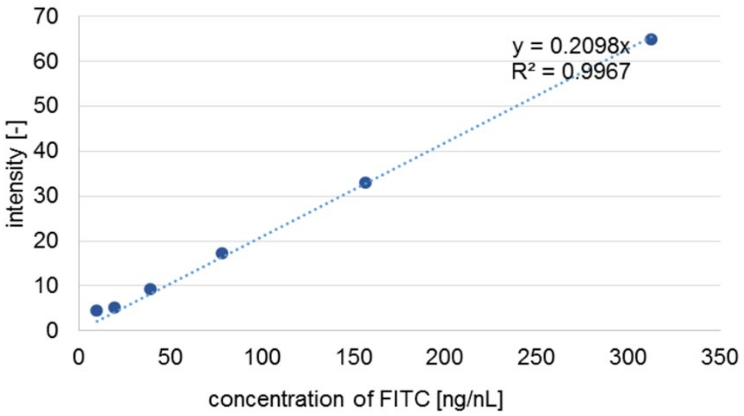

Figure S9. Calibration curve of concentration of FITC

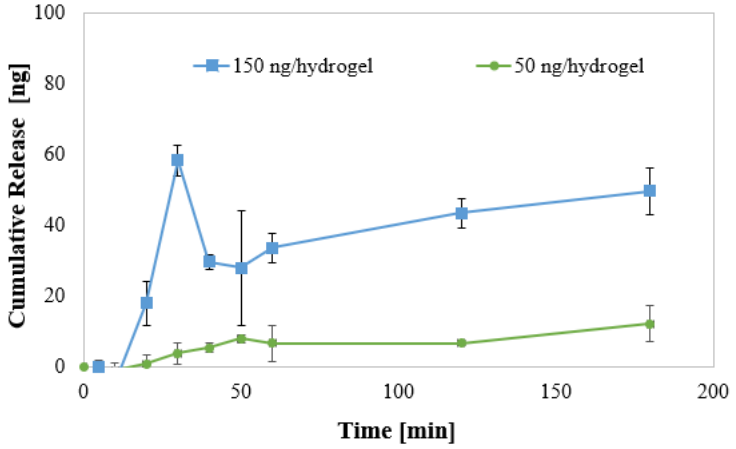

Figure S10. Profiles of FITC-bFGF release from FITC-bFGF loaded HA hydrogels

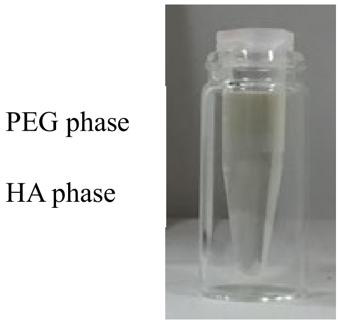

Figure S11. Results of distribution of FITC-bFGF in PEG/HA two phase system
